# MVNMDA: A Multi-View Network Combing Semantic and Global Features for Predicting miRNA–Disease Association

**DOI:** 10.3390/molecules29010230

**Published:** 2023-12-31

**Authors:** Chen Yang, Zhen Wang, Shanwen Zhang, Xiaoqian Li, Xuqi Wang, Jiashan Liu, Ruixiang Li, Sihao Zeng

**Affiliations:** School of Electronic Infomation, Xijing University, Xi’an 710123, China; yangchen0372@163.com (C.Y.); zhangshanwen@xijing.edu.cn (S.Z.); lxq980617@163.com (X.L.); wangxuqi@xijing.edu.cn (X.W.); bys.liu39@gmail.com (J.L.); lirx17735749731@163.com (R.L.); 18870126220@163.com (S.Z.)

**Keywords:** miRNA–disease association, multi-view network, cascade attention, meta-path

## Abstract

A growing body of experimental evidence suggests that microRNAs (miRNAs) are closely associated with specific human diseases and play critical roles in their development and progression. Therefore, identifying miRNA related to specific diseases is of great significance for disease screening and treatment. In the early stages, the identification of associations between miRNAs and diseases demanded laborious and time-consuming biological experiments that often carried a substantial risk of failure. With the exponential growth in the number of potential miRNA-disease association combinations, traditional biological experimental methods face difficulties in processing massive amounts of data. Hence, developing more efficient computational methods to predict possible miRNA-disease associations and prioritize them is particularly necessary. In recent years, numerous deep learning-based computational methods have been developed and have demonstrated excellent performance. However, most of these methods rely on external databases or tools to compute various auxiliary information. Unfortunately, these external databases or tools often cover only a limited portion of miRNAs and diseases, resulting in many miRNAs and diseases being unable to match with these computational methods. Therefore, there are certain limitations associated with the practical application of these methods. To overcome the above limitations, this study proposes a multi-view computational model called MVNMDA, which predicts potential miRNA-disease associations by integrating features of miRNA and diseases from local views, global views, and semantic views. Specifically, MVNMDA utilizes known association information to construct node initial features. Then, multiple networks are constructed based on known association to extract low-dimensional feature embedding of all nodes. Finally, a cascaded attention classifier is proposed to fuse features from coarse to fine, suppressing noise within the features and making precise predictions. To validate the effectiveness of the proposed method, extensive experiments were conducted on the HMDD v2.0 and HMDD v3.2 datasets. The experimental results demonstrate that MVNMDA achieves better performance compared to other computational methods. Additionally, the case study results further demonstrate the reliable predictive performance of MVNMDA.

## 1. Introductions

MicroRNA are endogenous non-coding RNA, approximately 20–24 nucleotides in length, that play a crucial regulatory role in numerous biological processes [1,2]. Since the discovery of the first miRNA in 1993, they have garnered significant attention from researchers, prompting investigations into their functions and mechanisms [3]. Recent studies have confirmed a causal relationship between miRNA dysregulation and the onset of diseases, with specific miRNA potentially acting as suppressors of certain cancers. For instance, miR-17-92 has been associated with diseases such as colorectal cancer, B-cell lymphoma, gastric cancer, and small-cell lung cancer [4,5,6]. The expression levels of miR-195 and miR-497 are inversely correlated with the malignancy of breast cancer, and research indicates their effectiveness in inhibiting cancer cell proliferation [7]. Furthermore, studies reveal that miRNA-19b is positively correlated with tumor size, miRNA-17a overexpression is associated with lymph node metastasis, and miRNA-18a expression is linked to tumor staging [8]. Therefore, unveiling more potential associations between miRNA and diseases is of paramount importance for understanding the mechanistic operations of diseases in the human body and for the development of more effective treatment strategies. Nevertheless, traditional biological experimental methods are often time-consuming, costly, and come with a significant risk of failure. There is an urgent need for a simple and efficient computational approach to predict disease-associated miRNA from a wealth of known associations and prioritize them for further validation and testing by researchers.

In recent years, numerous computational methods have been developed for predicting associations between miRNAs and diseases. Ji et al. [9] developed a computational method called AEMDA, a deep autoencoder with no negative samples, designed to identify potential associations between miRNAs and diseases. AEMDA derives dense and high-dimensional representations of diseases and miRNAs from disease semantic similarity, miRNA functional similarity, and heterogeneous interaction data. Subsequently, it utilizes reconstruction errors to predict disease-associated miRNAs. Wang et al. [10] proposed a computational method named SAEMDA. SAEMDA employs stacked autoencoders for unsupervised pre-training on all miRNA-disease samples. It then fine-tunes the model in a supervised learning manner using positive and negative samples. Its advantage lies in effectively utilizing information from both known and unknown samples. Zhao et al. [11] developed adaptive augmentation for miRNA-disease association prediction (ABMDA) to predict potential associations between diseases and miRNAs by balancing positive and negative samples using k-means clustering-based random sampling on negative samples. Similarly, the GBDT-LR proposed by Zhou et al. [12] used k-means clustering to screen out negative samples from unknown miRNA disease associations. The gradient boosting decision tree (GBDT) model is then used to extract features, as it has an inherent advantage in discovering many distinguishing features and feature combinations. Finally, the newly extracted features from the GBDT model are incorporated into a logistic regression (LR) model for predicting the final miRNA-disease association score.

Subsequently, influenced by the significant advances achieved by graph neural networks in other domains, several computational methods based on graph neural networks have been introduced. Li et al. [13] devised a novel algorithm based on two pieces of integrated similarity data and named it GAEMDA. By aggregating neighborhood information of nodes, GAEMDA obtains low-dimensional embeddings for miRNA and disease nodes. These embeddings are then fed into a bilinear decoder to identify potential associations between miRNAs and diseases. Ding et al. [14] introduced a variational graph autoencoder model (VGAEMDA) for predicting miRNA-disease associations. VGAEMDA derives feature expressions for miRNA and disease from a heterogeneous network, followed by calculating the miRNA-disease association probabilities using two variational graph autoencoders (VGAE). Furthermore, Lou et al. [15] developed a computational method that constructs multiple association networks from multiple information sources and obtains feature representations by combining neighborhood information from these networks. These multimodal feature representations are then fed into a multi-layer perceptron for predicting potential miRNA-disease associations. Most recent work has employed additional datasets or tools to compute various similarity information as Supplementary Data. However, many miRNAs or diseases lack such information, imposing limitations on the use of these computational methods. To address this issue, Qu et al. [16] proposed a computation method based on deep matrix factorization, which utilizes only known association information as an initial feature to predict unknown miRNA-disease associations, thereby mitigating the shortcomings of previous work.

Although some progress has been made in miRNA-disease association prediction tasks using the aforementioned computational methods, there are still limitations in previous models. Firstly, in most previous studies, external databases were utilized to calculate miRNA functional similarity, disease semantic similarity, and other auxiliary information as aids in predicting the association between miRNAs and diseases. However, many miRNAs and diseases lack such information, thus limiting the scalability of these methods. Secondly, some computational methods only focus on the direct associations between miRNAs and diseases, neglecting the complex interactions and network structures between them. This leads to the extraction of shallow features and a failure to fully utilize the rich structural information present in known miRNA-disease associations. Additionally, some GNN-based computational methods rely solely on local information from neighboring nodes in known associations without extracting higher-order information, resulting in insufficient structural information being extracted. To overcome the aforementioned limitations of existing methods, we propose a novel computational method named MVNMDA for predicting associations between miRNAs and diseases. The proposed model solely utilizes known association information as input, constructs multiple views, and learns embedding features of different types of nodes. The architecture of the MVNMDA model consists of the following four components: (1) a local view based on known association information, employing graph convolution networks to aggregate features of first-order neighboring nodes; (2) a global view based on the assumption of full connectivity, aiming to mitigate the limitation of graph convolution networks, which rely solely on local relationships and lack the capability to aggregate node features at a higher level view; (3) a semantic view that captures semantic relationship information between different types of nodes in the heterogeneous graph through metapaths; (4) a cascaded attention classifier, which fuses multi-layer features through cascaded attention to reveal inter-layer and inter-feature correlations, suppress noise in features, and predict the probability of miRNA-disease associations.

1. A general framework that does not require extra data is proposed for predicting associations between miRNA and disease. This framework effectively extracts and integrates embedding between miRNA and disease from multiple views.
2. Based on predefined metapaths, miRNA–miRNA networks and disease–disease networks are constructed to complement the embedding information of different nodes. Unlike similarity-based computational methods, metapaths can illustrate how two entities are connected through specific semantic paths, thereby mining rich semantic information within the network.
3. We devised a cascaded attention classifier designed to eliminate redundant information and progressively integrate features, thereby obtaining more precise feature representations.

## 2. Results

### 2.1. Cross-Validation and Evaluation Metrics

Cross-validation is an essential method used to evaluate and statistically analyze models. It involves partitioning the dataset into multiple subsets, allowing the model to be trained and tested on different subsets multiple times. This approach enables a more comprehensive and objective assessment of the performance of the model. Taking five-fold cross-validation as an example, the dataset is divided into five equally sized subsets, with four of them used for model training, and the remaining one employed for model testing. Five-fold cross-validation is repeated five times, with a different subset chosen as the testing set in each iteration while the others serve as the training sets. Cross-validation is instrumental in reducing the randomness of model performance evaluations, enhancing the reliability of the assessment results. Furthermore, every sample in the dataset is included in both training and testing during cross-validation, thereby maximizing the utilization of available data, particularly when dealing with limited datasets. By considering various combinations of training and testing sets, a more comprehensive evaluation of the model generalization performance is attained.

Six common evaluation metrics are employed to assess the performance of models, including the area under the ROC curve (AUC), the area under the precision-recall curve (AUPR), accuracy, precision, recall, and F1 score. In the ROC curve, the x-axis represents the false positive rate (FPR), and the y-axis represents the true positive rate (TPR). The ROC curve is constructed based on different thresholds and their corresponding FPR and TPR values. In contrast, the PR curve depicts the relationship between the precision and recall metrics at different threshold settings. Both AUC and AUPR serve as two crucial indicators for evaluating the effectiveness of prediction models, with larger values indicating better model performance. The calculations for each metric are as follows:

(1) $$Accuracy=\frac{TP+FN}{TP+TN+FP+FN}$$

(2) $$Precision=\frac{TP}{TP+FP}$$

(3) $$Recall=\frac{TP}{TP+FN}$$

(4) $$F1=2\times \frac{Precision\times Recall}{Precision+Recall}$$

where TP is the number of correctly identified positive samples, and FN is the number of incorrectly identified positive samples. FP represents the number of falsely identified negative samples, and TN represents the number of correctly identified negative samples.

### 2.2. Experiment Setup

In this experiment, we constructed the MVNMDA using the PyTorch deep learning framework and the PyG graph deep learning framework. The latent feature dimension of the graph autoencoder, in different views, was set to 128, the training epoch was set to 30, and the training batch size was set to 256. To optimize the model, we chose the Adam optimizer and set the learning rate to 1e-5. Furthermore, referring to previous work [15], the HMDD v2.0 [17] and HMDD v3.2 [18] datasets were obtained. To mitigate the impact of overfitting and computational errors, 5-fold and 10-fold cross-validation were employed on both datasets to train and evaluate the model. In the 5-fold cross-validation experiment, an equal number of negative samples as positive samples were randomly selected from unknown associations, and these samples were then divided into five mutually exclusive subsets. In each experiment, one subset was used as the test set, while the remaining four subsets comprised the training set. Similarly, in 10-fold cross-validation, all samples were evenly divided into ten mutually exclusive subsets to ensure the accuracy and reliability of the validation results.

### 2.3. Comparison with Other Methods

In the section, we compare the proposed MVNMDA with twelve other computational models, namely, AEMDA [9], ABMDA [11], GBDT-LR [12], EDTMDA [19], VAEMDA [20], NIMCGCN [21], ERMDA [22], GAEMDA [13], SAEMDA [10], VGAEMDA [14], MINIMDA [15], and GCNMDA [23].

- AEMDA initially extracts high-dimensional and high-density features of miRNAs and diseases. Subsequently, the reconstruction error is utilized to represent the probability of miRNA-disease associations. Furthermore, AEMDA is a computational method that does not require negative samples.
- ABMDA generates balanced positive and negative samples and employs a decision tree classifier to infer the associations between miRNAs and diseases.
- GBDT-LR employs the gradient boosting decision tree (GBDT) model to obtain effective embeddings, which are then utilized to predict the final miRNA-disease association scores through a logistic regression (LR) model.
- EDTMDA comprehensively learns the embeddings of miRNA–disease pairs through the integration of statistical metrics, graph-theoretical measures, and matrix factorization results.
- VAEMDA trains the variational autoencoder (VAE) based on two spliced matrices and obtains final predicted association scores between miRNAs and diseases obtained by integrating the scores from the two trained VAE models.
- NIMCGCN employed a graph convolutional network (GCN) to learn latent feature representations of miRNAs and diseases from two similarity networks and then applied a neural inductive matrix completion (NIMC) model to predict their latent association.
- ERMDA is the ensemble learning method to learn the feature representation of miRNAs and disease by integrating similarity from multiple balanced training subsets and using multiple independent learners to predict miRNA-disease associations jointly.
- GAEMDA employs graph autoencoders to learn the low-dimensional embeddings of miRNAs and diseases. Subsequently, these embeddings of miRNA and disease nodes are fed into a bilinear decoder to identify the potential associations between miRNAs and diseases.
- SAEMDA initially pretrains the stacked autoencoders in an unsupervised manner. Subsequently, it fine-tunes them in a supervised approach to predict potential associations.
- VGAEMDA obtains the features of miRNAs and diseases from the heterogeneous network, and subsequently utilizes two variational graph autoencoders (VGAE) to compute the miRNA-disease association scores.
- MINIMDA is a computational approach that constructs multiple information networks leveraging multiple sources of information. This network neighborhood information is then blended to derive the feature representations of miRNAs and diseases.
- GCNMDA utilizes graph convolutional networks (GCN) to extract deeply embedded features of miRNAs and diseases. These embedded features are then processed using multi-layer perceptrons (MLP) by GCNMDA to predict the association probabilities between miRNAs and diseases.

**Figure 1:**
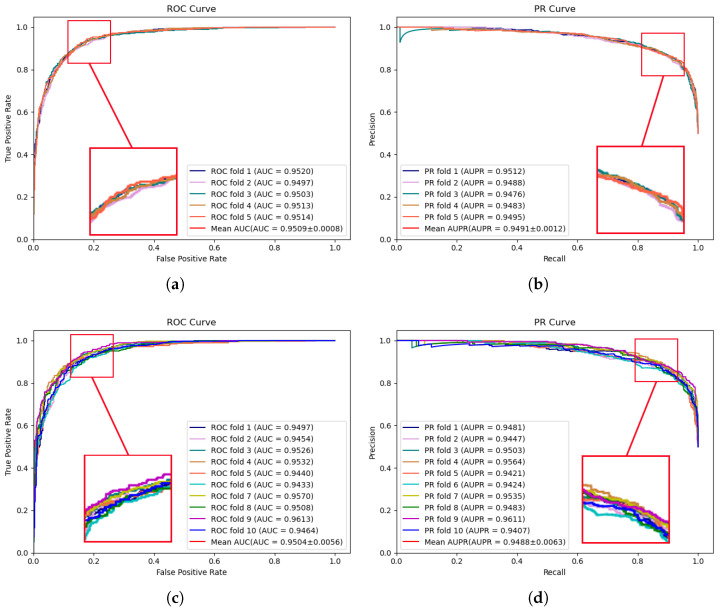
ROC and PR curves performed by MVNMDA on the HMDD v2.0 dataset. (**a**,**b**) The performance of the proposed MVNMDA in terms of ROC curve and PR curve based on 5-fold CV. (**c**,**d**) The performance of the proposed MVNMDA in terms of the ROC curve and PR curve based on 10-fold CV.

To comprehensively assess the predictive performance of the proposed model and the compared models, 5-fold and 10-fold cross-validation are conducted on two publicly available datasets, HMDD v2.0 [17] and HMDD v3.2 [18], respectively. These extensive experiments facilitate a more comprehensive evaluation of the model performance across diverse data subsets, thereby enhancing the robustness and reliability of the validation results. Figure 1 illustrates the performance results of the proposed model on the HMDD v2.0 dataset. In 5-fold cross-validation, an AUC of 0.9509 and an AUPR of 0.9491 were achieved by MVNMDA. In the case of 10-fold cross-validation, similar precision was exhibited by MVNMDA, with an AUC value of 0.9504 and an AUPR value of 0.9488. These results highlight the robustness of MVNMDA under different cross-validation settings. A detailed performance comparison between MVNMDA and other methods in both 5-fold and 10-fold cross-validation is presented in Table 1. The findings indicate that the proposed method outperformed other models in terms of AUC, AUPR, and precision metrics. Specifically, in 5-fold cross-validation, the proposed model achieved an AUC of 0.9509, representing a 0.65 improvement over the second-best model (MINIMDA). The AUPR metric reached 0.9491, which was 1.28 higher than the second-best model (VAGEMDA). The accuracy metric attained the second-best value of 0.8768, slightly lower than the best model (MINIMDA) by 0.2. The precision metric was 0.8651, demonstrating a 1.05 improvement over the second-best model (MINIMDA). In ten-fold cross-validation, MVNMDA attained an AUC of 0.9504, showing a 0.98 improvement over the second-best model (MINIMDA). The AUPR metric was 0.9488, marking a 1.27 increase over the second-best model. The accuracy metric reached 0.8787, representing a 0.4 improvement over the second-best model. The precision metric was 0.8828, exhibiting an increase of 3 over the second-best model (ERMDA).

**Figure 2:**
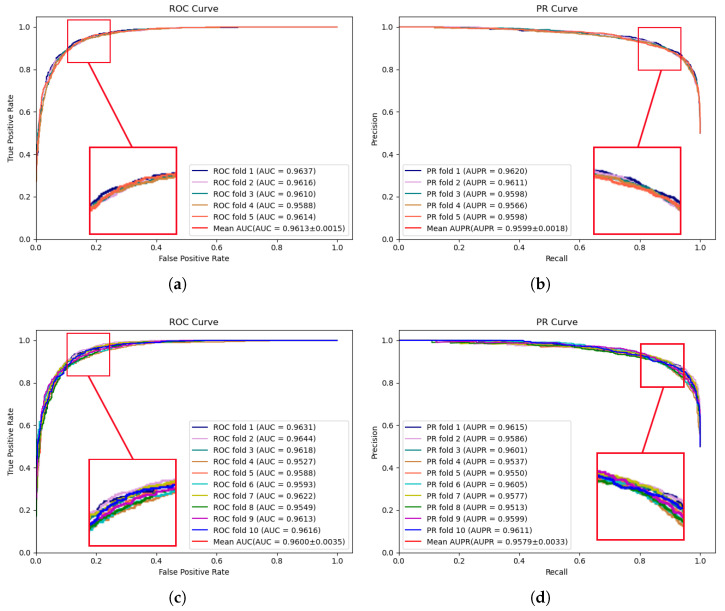
ROC and PR curves performed by MVNMDA on HMDD v3.2 dataset. (**a**,**b**) The performance of the proposed MVNMDA in terms of the ROC curve and PR curves based on 5-fold CV. (**c**,**d**) The performance of proposed MVNMDA in terms of the ROC curve and PR curve based on 10-fold CV.

The experimental results of MVNMDA on the HMDD v3.2 dataset are depicted in Figure 2. The results demonstrate that during 5-fold cross-validation, MVNMDA achieved an AUC of 0.9613 and an AUPR of 0.9599. With 10-fold cross-validation, it yielded an AUC value of 0.9600 and an AUPR value of 0.9579. These experimental findings serve to further substantiate the resilience of the proposed model across various datasets.

**Figure 3:**
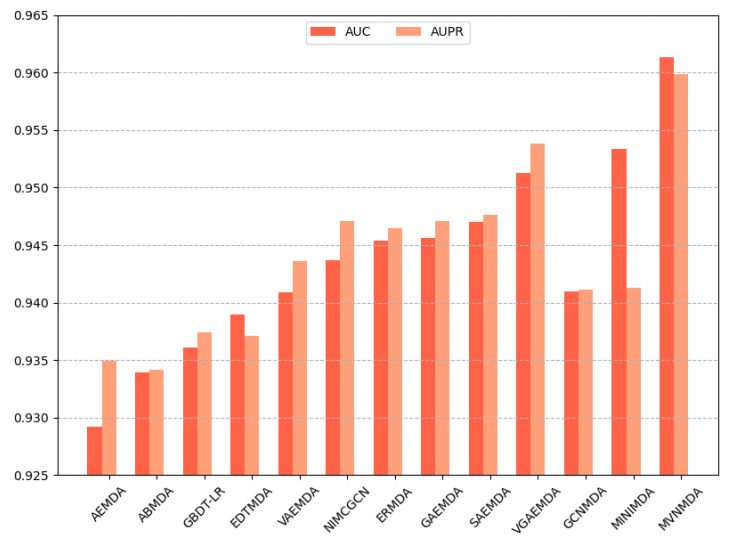
The performance comparison of different methods on the HMDD v3.2 dataset by 5-CV.

**Figure 4:**
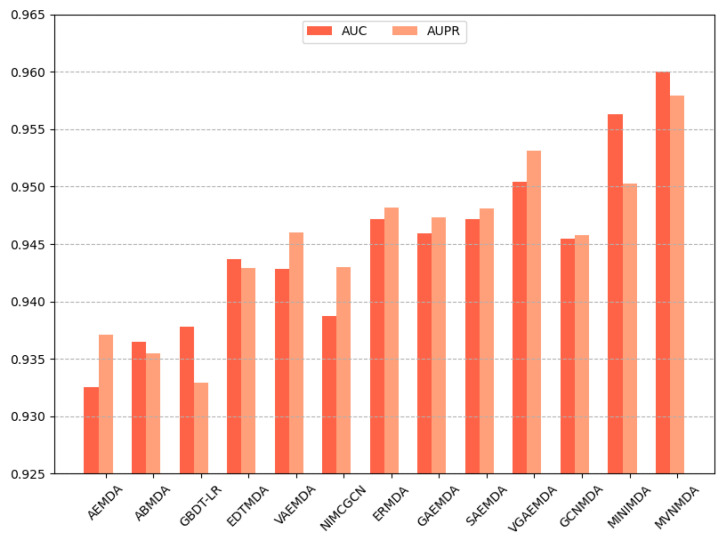
The performance comparison of different methods on the HMDD v3.2 dataset by 10-CV.

We opted for AUC and AUPR as the primary performance metrics when comparing the proposed model with other models on the HMDD v3.2 dataset to assess the predictive accuracy of different models. In the 5-fold cross-validation, as illustrated in Figure 3, the proposed model outperformed its counterparts in both the AUC and AUPR metrics. The AUC metric exhibits a 0.79 improvement compared to the second-best model (MINIMDA), while the AUPR metric shows a 0.61 increase compared to the second-best model (VGAEMDA). The comparative results for 10-fold cross-validation are presented in Figure 4, where the MVNMDA model surpassed the second-best model (MINIMDA) by 0.37 in AUC and improved AUPR by 0.48 compared to the second-best model (VGAEMDA).

### 2.4. Ablation Studies

In this subsection, ablation experiments are conducted to evaluate the contributions of each module within the proposed model. We also investigate whether the classifier incorporates the cascaded attention module. In MVNMDA, node feature updates encompass three types of views: local view, global view, and semantic view. These views differ in their receptive fields. The local view entails that, during message propagation in the graph convolutional layer, only first-order neighbors are taken into consideration, rendering this operation inherently local [24]. The global view posits that every node in the graph is fully interconnected, with the aim of extracting more advanced features. The semantic view entails the generation of multiple walk-paths based on known association information, and message propagation and aggregation rely on the association information furnished by these paths. Table 2 provides the results of the ablation experiments for different views and the cascaded attention.

Table 2 presents an intuitive comparison of the contributions of different views to predictions. The results demonstrate that, despite the localized nature of the local view, it remains a predominant contributor to predictions. In the presence of the cascaded attention module, the MVNMDA model attains an AUC value of 0.9566 and an AUPR value of 0.9528. This achievement surpasses previous work and can be attributed to the role of the GCN-MLP block of the graph autoencoder in the local view, effectively addressing over-smoothing issues. Without cascaded attention, the AUC and AUPR values are 0.9555 and 0.9524, respectively, indicating an acceptable decrease in performance. Furthermore, the local view with cascaded attention yields improvements in accuracy, recall, and F1 scores, affirming the effectiveness of cascaded attention.

In addition, the results show that the performance of the semantic view alone is inferior to the local view. The AUC and AUPR values of the semantic view with cascaded attention are 0.9424 and 0.9427, while the values of the semantic view without cascaded attention are 0.9382 and 0.9389, indicating performance differences of 0.0042 and 0.0038, further validating the cascaded attention module.

The subsequent analysis focuses on the performance of combining two types of views, which include (1) the fusion of the local and global views, (2) the fusion of the local and semantic views, and (3) the fusion of the global and semantic views. In the presence of cascaded attention, (1) and (2) show no significant differences in AUC and AUPR scores. In the absence of cascaded attention, the differences in AUC and AUPR between (1) and (2) are 0.0024 and 0.0035, while (3) exhibits relatively poorer performance.

After the fusion of all views, models with cascaded attention achieve AUC and AUPR values of 0.9613 and 0.9599, while models without cascaded attention have values of 0.9587 and 0.9568, both achieving the best results. The comparative results indicate that the local view dominates predictions, while the global view and semantic view can serve as non-local complementary information to enhance the model’s predictive accuracy.

### 2.5. Parameter Analysis

Hyperparameters are essential elements in deep learning models, and they can significantly influence the performance of a model. In this study, the selection of hyperparameters is equally critical and can have a substantial impact on prediction accuracy and stability. Therefore, to ensure the optimal performance of MVNMDA, we conducted a 5-cv experiment on the HMDD v3.2 dataset, carefully exploring four hyperparameters: feature embedding dimension, training epoch, batch size, and learning rate.

**Figure 5:**
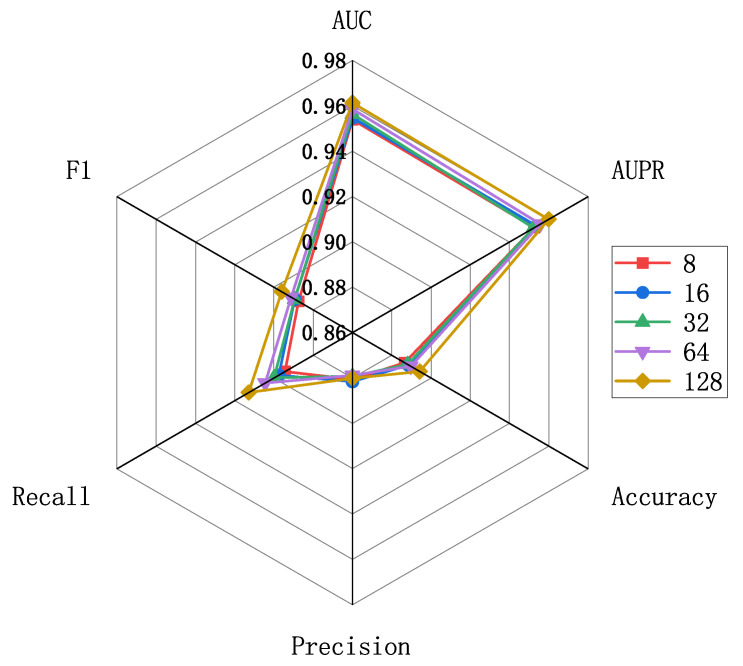
Parameter analysis for feature embedding dimensions.

**Figure 6:**
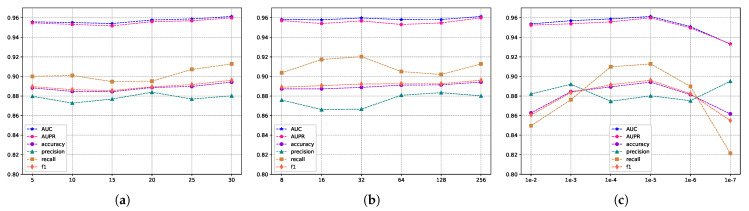
The metric scores under different hyperparameters. (**a**) The impact of training epochs on performance. (**b**) The influence of batch sizes on performance, and (**c**) the effect of different learning rates on performance.

The evaluation scores for different embedding feature dimensions of miRNA and diseases are illustrated in Figure 5, where the embedding feature dimensions are {8, 16, 32, 64, 128}. As indicated by Figure 5, variations exist in the results across different embedding feature dimensions, particularly in terms of recall and F1 metrics. To ensure both model accuracy and computational efficiency, we set the embedding feature dimensions for miRNA and disease nodes to 128 in this experiment. This decision is grounded in the analysis and trade-off considerations of evaluation metrics, aiming to strike a balance between model performance and computational efficiency. Furthermore, we conducted an independent exploration of the train epoch, batch size, and learning rate. Figure 6a displays the scores across various metrics as the train epoch parameter varies {5, 15, 20, 25, 30}. The results indicate that, although the model exhibits some minor fluctuations, it is generally insensitive to this parameter. Subsequently, Figure 6b demonstrates the performance differences for different batch sizes {8, 16, 32, 64, 128, 256}. The scores for AUC and AUPR metrics remain stable, suggesting that batch size has a minimal impact on the model’s performance. Finally, Figure 6c reveals the model’s sensitivity to the learning rate, spanning {1e-2, 1e-3, 1e-4, 1e-5, 1e-6, 1e-7}. The model shows greater sensitivity to this parameter, with the highest scores for AUC and AUPR metrics achieved when the learning rate is set to 1e-5. Performance sharply declines when the learning rate is set below 1e-5. In conclusion, based on the experimental results of parameter analysis, the embedding feature dimensions for miRNA and disease nodes were set to 128, while the Train epoch was set to 30. Additionally, the batch size was set to 256, and the learning rate was set to 1e-5 in order to achieve optimal model performance.

### 2.6. Case Studies

To assess the predictive capabilities of MVNMDA for unknown miRNA-disease associations, we conducted case studies involving three diseases: breast cancer, esophageal cancer, and lung cancer. Specifically, the model is trained using all known associations from the HMDD v2.0 dataset. Following the training phase, the model is employed to estimate the probability of associations between all miRNAs and specific diseases. Subsequently, the known associations were removed, and the prediction results were ranked in descending order. The top 30 candidate miRNAs, based on their prediction scores, were subjected to validation using the most recent HMDD database and the dbDEMC database [25].

The first case study is breast cancer, a malignant tumor and a significant health concern for women worldwide. It primarily affects the breast tissue in females. Although men can also develop breast cancer, their incidence is notably lower than in women. It is estimated that there will be over 2.26 million new cases and 685,000 deaths in 2020 [26]. The incidence and mortality rates of breast cancer are closely associated with the level of development in a given region, with higher-developed countries showing significantly improved 5-year survival rates compared to lower-developed nations. Therefore, despite the rapid increase in the global incidence of breast cancer, relatively high mortality is not inevitable. Regular breast cancer screenings and self-examinations are pivotal in early detection, with early discovery and timely treatment effectively reducing patient mortality rates [27,28]. Table 3 presents the candidate miRNAs predicted by MVNMDA related to breast cancer, and among the top 30 predicted results, 29 miRNAs have been validated using the latest HMDD database, resulting in a prediction accuracy of 96.66%.

The second case study is esophageal cancer, which is a malignant tumor. The esophagus is the tube that connects the throat to the stomach and is responsible for transporting ingested food from the throat to the stomach for digestion. Esophageal cancer typically forms cancer cells in the cells lining the inner wall of the esophagus. It may initially appear as small ulcers or polyps and gradually grow and spread [29]. Table 4 presents the prediction results of the esophageal cancer. Among the top 30 miRNAs predicted by the model, 28 have been validated using the latest HMDD database.

The third case study is lung cancer, one of the most common and deadliest types of cancer worldwide [30]. Lung cancer is closely associated with factors such as smoking and air pollution. It often exhibits no obvious symptoms in the early stages, but as the tumor grows, patients may experience chronic cough, coughing up blood-streaked sputum, difficulty breathing, chest pain, hoarseness, sore throat, and recurrent infections. MVNMDA predicts unknown miRNAs related to lung cancer based on known miRNA-disease associations and validates them using additional datasets. The prediction results are presented in Table 5. Among the top 30 results, 28 have been validated through HMDD3.2.

### 2.7. Survival Analysis

**Figure 7:**
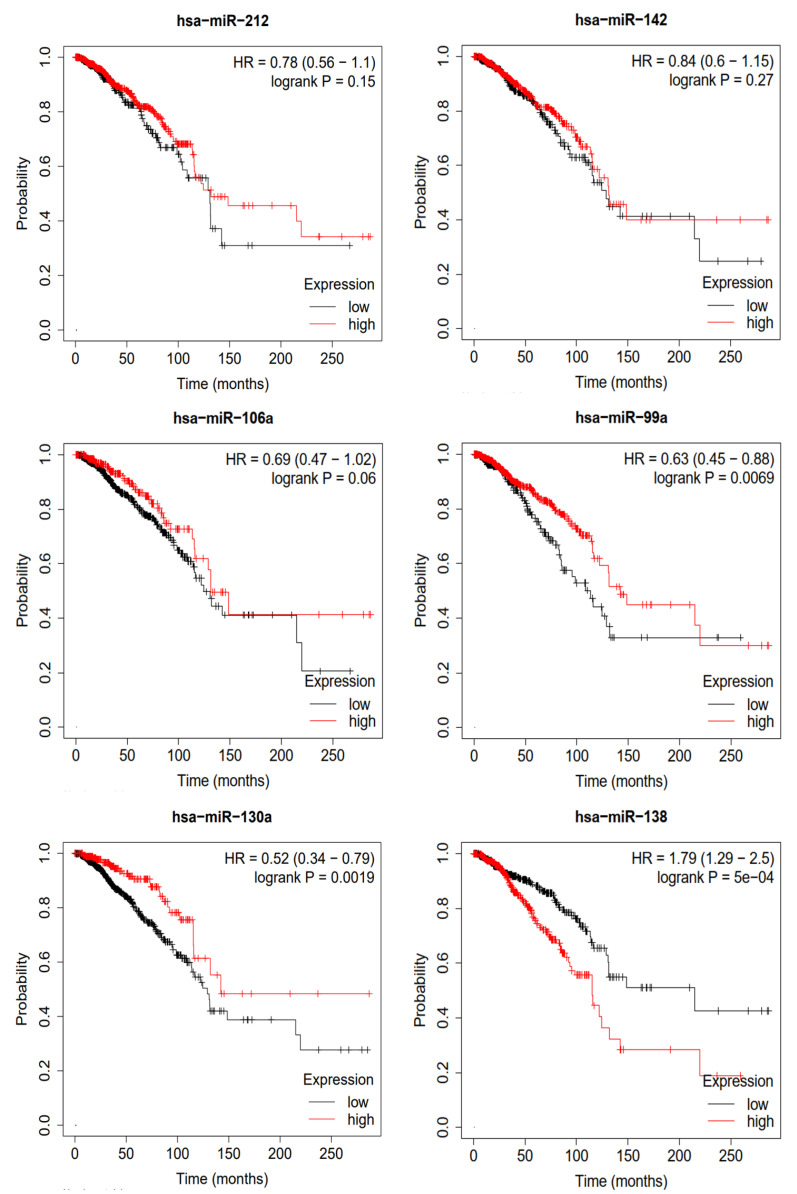
Survival analysis of top 6 predictive miRNA in breast cancer.

To validate the miRNAs predicted by MVNMDA and obtain confirmation from clinical data, we conducted a Kaplan–Meier survival analysis using clinical data from breast cancer sourced from the Cancer Genome Atlas (TCGA) database [31]. This analysis aimed to assess the prognostic value of these miRNAs in breast cancer patients, and the results are depicted in Figure 7. We selected the top six miRNAs with the highest predicted scores for breast cancer by MVNMDA, namely miRNA-212, miRNA-142, miRNA-106a, miRNA-99a, miRNA-130a, and miRNA-138. Specifically, breast cancer patients with higher expression levels of miRNA-212 and miRNA-142 exhibit a slightly higher overall survival rate compared to those with lower expression levels. Furthermore, overexpression of miRNA-106a and miRNA-99a is positively correlated with the overall survival rate, as patients with overexpression have higher survival rates than those with lower expression levels. Studies have suggested that miRNA-99a acts as a suppressor in various cancers, including breast cancer [32]. Overexpression of miRNA-99a exerts multiple inhibitory effects on breast cancer cells, reducing their vitality and inhibiting proliferation and metastasis, potentially slowing down tumor growth. On the other hand, miRNA-99a induces apoptosis in cancer cells, aiding in the removal of abnormal cells. This highlights the critical role of miRNA-99a in impeding the development of breast cancer, making it a potential therapeutic target to improve patient survival rates. Patients with higher miRNA-130a expression levels are more likely to have a better prognosis. Further research has indicated that miRNA-130a plays a role in suppressing the migration and invasion of breast cancer cells [33]. This miRNA effectively inhibits the migration and invasion of breast cancer cells, limiting tumor spread and metastasis. MiRNA-130a shows promise in breast cancer research and treatment, potentially offering improved survival and recovery opportunities for breast cancer patients. However, overexpression of miRNA-138 leads to a significant decrease in the overall survival rate of breast cancer patients. As shown in Table 6, patients with lower expression levels have a median survival period of 215.2 months, whereas those with overexpression have a median survival period of only 115.4 months.

The results of the survival analysis using breast cancer cases indicate that the miRNA predicted by the proposed model, which is associated with breast cancer, may play a significant role in early prevention, early diagnosis, and prognosis assessment of breast cancer. This suggests that these miRNAs hold the potential to provide support in breast cancer research and treatment, offering valuable information for physicians to develop more personalized treatment plans. Additionally, this underscores the potential of deep learning in the field of biomedical science, as it can assist in better understanding disease mechanisms and improving early disease diagnosis and treatment outcomes.

## 3. Discussion

The experimental results demonstrate that MVNMDA exhibits better performance compared to twelve other computational methods. We conducted a thorough analysis of the impact of different feature combinations and dimensionality settings on the results. Furthermore, through case studies on three specific diseases, we validated the accuracy of the proposed method and provided strong support using clinical data from the TCGA dataset. Overall, MVNMDA significantly improves prediction performance by extracting non-linear representations of miRNAs and diseases through multi-view learning and fusing these features using a cascade attention and based on known miRNA-disease network information to more comprehensively reveal the complex associations between miRNAs and diseases. However, there are limitations to our work. Firstly, the limited number of experimentally validated miRNA-disease associations and the difficulty in determining whether a specific miRNA is not associated with a particular disease pose challenges in selecting negative samples. Secondly, for isolated miRNA or disease nodes lacking known association information, effectively learning their embedded features is challenging. Additionally, known association networks are often discrete and incomplete, which can bias the model towards nodes with more associations during feature extraction, overlooking nodes with fewer associations and reducing prediction accuracy for specific miRNAs or diseases. Future research can further explore more effective strategies for selecting negative samples and addressing isolated nodes using methods such as unsupervised learning. Simultaneously, incorporating advanced techniques such as graph neural networks can be considered to more comprehensively capture complex patterns in association networks, thereby enhancing the predictive performance of the model.

## 4. Materials and Methods

### 4.1. Datasets

The Human microRNA Disease Database (HMDD) is a database used for studying the associations between human miRNAs and diseases. It collects a substantial amount of experimentally verified data, including information regarding the correlation between miRNAs and various diseases. Following previous work, we have downloaded two benchmark datasets, namely HMDD v2.0 and HMDD v3.2 (https://github.com/chengxu123/MINIMDA (accessed on 13 December 2023)). Among these, the HMDD v2.0 dataset comprises 5430 associations between human miRNAs and diseases, encompassing 495 miRNAs and 383 distinct diseases. The HMDD v3.2 dataset contains 8968 associations, involving 788 miRNAs and 374 different diseases. More detailed information is provided in Table 7. Based on this information, the association network of miRNA-disease can be represented using an adjacency matrix *A*, where *m* is the number of miRNAs, *n* is the number of diseases. If a known association exists between miRNA($m_{i}$) and disease($d_{j}$), then $A_{ij}=1$; otherwise, $A_{ij}=0$.

### 4.2. MVNMDA

**Figure 8:**
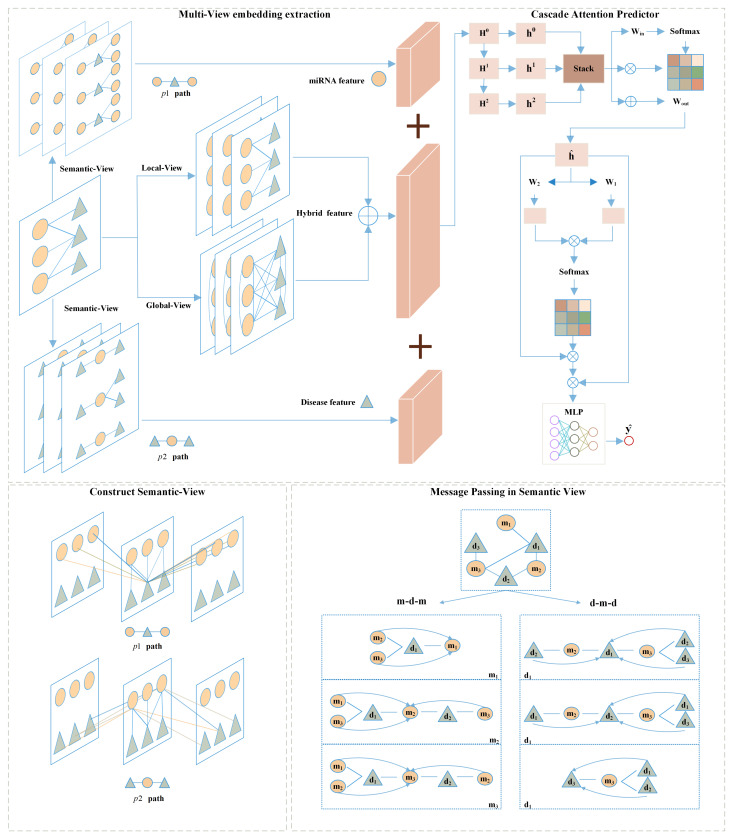
The framework of our proposed MVNMDA for predicting miRNA-disease association.

In this subsection, we will first outline the proposed MVNMDA architecture and then provide a comprehensive overview of each component. As depicted in Figure 8, MVNMDA employs multiple views, including the local view, global view, and semantic view, to acquire embedded features for all nodes. Subsequently, a coarse-to-fine approach is employed, utilizing a cascade attention predictor to learn composite features for various node types. The cascade attention predictor comprises the layer correlation module, feature correlation module, and prediction head, each responsible for focusing on interrelations among multi-layer features, refining relationships between feature elements, and making association predictions. In the following, we will delve into the detailed computational processes of each module.

#### 4.2.1. Local-View

In the local-view, we employ a graph convolutional network (GCN) as an encoder to learn low-dimensional embeddings of all nodes. The decoder is used to reconstruct the structural information, namely the graph adjacency matrix. Subsequently, the graph autoencoder is optimized by minimizing the gap between the real adjacency matrix and the reconstructed adjacency matrix.

The GCN takes the graph as input and aggregates the neighboring embedding features of each node based on the structure of the graph. This aggregation strategy for node features enables the GCN to efficiently learn both the features of nodes within the graph and the association relationships between these nodes. GCN and its variations have found widespread applications in various biological link prediction tasks, significantly enhancing prediction accuracy. These applications include predicting miRNA-disease associations and drug affinity, as documented in previous studies [34,35,36]. The graph autoencoder utilizes known associations as the structure of the graph to aggregate neighboring nodes and generate node embedding features with local receptive fields. To obtain the embedding for each node based on the graph’s structural information, the adjacency matrix $A_{local}\in R^{\left(m+n)\times (m+n\right)}$ is described as follows:

(5) $$A_{local}=\left(\begin{array}{cc} E_{m} & A\\ A^{T} & E_{n} \end{array}\right)$$

where $A^{T}$ is the transpose of known association matrix $A\in R^{m\times n}$. $E_{m}$ and $E_{n}$ is the identity matrix of m×m and n×n, respectively. Then, we adopt GCN to extract the node embedding in $A_{local}$. Meanwhile, in order to mitigate the over-smoothing problem, we combined the GCN layer with a fully connected layer, defined as:

(6) $$H^{l}=\sigma \left(GCN\left(A_{local},H^{l-1}\right)+MLP\left(H^{l-1}\right)\right)$$

(7) $$GCN\left(A_{local},H^{l-1}\right)=\sigma \left({\tilde{D}}_{local}^{\frac{1}{2}}{\tilde{A}}_{local}{\tilde{D}}_{local}^{\frac{1}{2}}H^{l-1}W^{l-1}\right)$$

**Figure 9:**
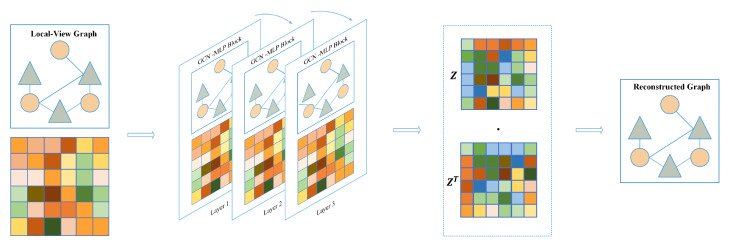
Graph autoencoder based on local-view.

In Equation (3), it is noted that ${\tilde{A}}_{local}=I+A_{local}$, where *I* is the identity matrix, $\tilde{D}$ is the degree matrix of $A_{local}$, and $W^{l-1}$ denotes the trainable weight matrix. GCN takes node embedding $H^{l-1}$ of the previous layer to compute embedding $H^{l}$ of the current layer. In order to mitigate the over-smoothing problem, we introduce a multi-layer perceptron to assist GCN in extracting embedding features. Lastly, the ReLU nonlinear function is adopted to activate the latent embedding feature, and the flowchart of the local-view graph autoencoder is shown in Figure 9.

The encoder in our study consists of three layers of graph convolutional network–multi layer perceptron (GCN-MLP) blocks. The decoder utilizes inner product operation between latent embedding features for identifying potential associations, defined as:

(8) $${\hat{A}}_{local}=\sigma \left(z\cdot z^{T}\right)$$

(9) $$Loss_{local}=\frac{1}{N}\sum_{i=1}^{n}\left(A_{local}log\left({\hat{A}}_{local}\right)+\left(1-A_{local}\right)log\left(1-{\hat{A}}_{local}\right)\right)$$

where *z* is the last layer node embedding of the encoder and $y_{local}$ denotes the reconstructed adjacency matrix. During training, the model is optimized by minimizing the loss between the real and reconstructed adjacency matrix.

#### 4.2.2. Global-View

In contrast to the local-view, the global-view assumes that known graph structures are fully connected graphs, meaning that each node is connected to other nodes. Therefore, we construct a based global-view graph adjacent matrix $A_{global}\in 1^{\left(m+n)\times (m+n\right)}$ filled with scalar value 1.

**Figure 10:**
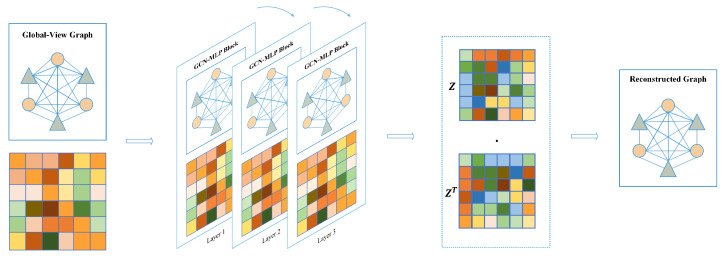
Graph autoencoder based on global-view.

As shown in Figure 10, same as for the local view, we stacked GCN-MLP block as the encoder to extract the embedding features of all nodes based on the fully connected graph, defined as:

(10) $$H^{l}=\sigma \left(GCN\left(A_{global},H^{l-1}\right)+MLP\left(H^{l-1}\right)\right)$$

(11) $$GCN\left(A_{global},H^{l-1}\right)=\sigma \left({\tilde{D}}_{global}^{\frac{1}{2}}{\tilde{A}}_{global}{\tilde{D}}_{global}^{\frac{1}{2}}H^{l-1}W^{l-1}\right)$$

Similarly, in Equation (6), ${\tilde{A}}_{global}$ denotes the adjacency matrix with identity matrix I added. $D_{global}$ is the degree matrix of ${\tilde{A}}_{global}$, and $W^{l-1}$ denotes a learnable weight matrix. After three layers of GCN-MLP Block computation, the non-local node feature $H^{3}$ is obtained and used as latent feature *z*. Subsequently, we use latent features to reconstruct the adjacency matrix and iteratively supervise the loss between the reconstructed adjacency matrix and the true adjacency matrix, which is calculated as follows:

(12) $${\hat{A}}_{global}=\sigma \left(z\cdot z^{T}\right)$$

(13) $$Loss_{local}=\frac{1}{N}\sum_{i=1}^{n}\left(A_{global}log\left({\hat{A}}_{global}\right)+\left(1-A_{global}\right)log\left(1-{\hat{A}}_{global}\right)\right)$$

#### 4.2.3. Semantic-View

A meta-path is a walking path that contains multiple node types and is denoted in the form of $\left\{A_{1}-A_{2}-\ldots -A_{n}\right\}$, where start node $A_{1}$ and end node $A_{n}$ are the same type. A meta-path can better model the task in the real world and can better help to analyze and understand the semantic information in the complicated network [37].

Two examples of meta-paths are presented in Figure 9, which represent different semantic information by a sequence of nodes in the path. The existing message-passing mechanism cannot directly capture the implicit relation between nodes of the path, but the meta-path can provide the implicit link so that the features can be updated by the message-passing mechanism. For a detailed explanation, take miRNA-disease association prediction as an example. Given a set of miRNA node $M=\{m_{1},m_{2},\ldots ,m_{M}\}$ and a set of disease node $D=\{d_{1},d_{2},\ldots ,d_{D}\}$, the validated miRNA-disease association is defined $A\in {0,1}^{M\times N}$, where $A_{ij}=1$ if *i*th miRNA $m_{i}$ is associated with *j*th disease $d_{j}$, otherwise $A_{ij}=0$. Subsequently, we define $p_{1}$ as a meta-path denoted as (m−d−m) and $p_{2}$ as another meta-path denoted as (d−m−d), where *m* and *d* represent miRNA nodes and disease nodes, respectively.

In the semantic view provided by the metapaths, when updating node features in the graph, for any node $v_{i}$, it first obtains a subgraph formed by the walk-path and then uses the GCN-MLP Block to perform message passing and aggregation on the features of node $v_{i}$ based on the subgraph. Taking node $m_{1}$ in the $p_{1}$ path as an example, the metapath $p_{1}$ constructs implicit relationships with $m_{2}$ and $m_{3}$. When performing message passing, $m_{1}$ no longer relies solely on the features of $d_{1}$ nodes, expanding the receptive field. This allows the model to better extract complex relationships and information transfer between nodes, thereby improving performance and representational capability, contributing to a more comprehensive and accurate understanding of complex relationships and data.

#### 4.2.4. Cascade Attention Predictor

After obtaining low-dimensional feature representations from different types of views, we employed a cascaded attention mechanism to fuse features from shallow to deep layers. In the neural network, shallow-level feature vectors are often redundant. To preserve essential layer-specific information, a hierarchical attention mechanism is applied to assign distinct weights to different layers and facilitate inter-layer information exchange, with the aim of filtering out redundant information and obtaining high-quality semantic representations. Simultaneously, feature attention is employed to suppress noise among deep-layer semantic feature elements. The process of the cascade attention predictor is as follows:

(14) $$F=Concat(z_{mdm}^{m_{i}},z_{local}^{m_{i},d_{j}}+z_{global}^{m_{i},d_{j}},z_{dmd}^{d_{j}})$$

where $z_{mdm}^{m_{i}}$ represents the semantic features of miRNA ($m_{i}$) extracted through the metapath $p_{1}=\left\{\left(m,d,m\right)|m\in M,d\in D\right\}$, $z_{local}^{m_{i},d_{j}}$ and $z_{global}^{m_{i},d_{j}}$ represent the features of miRNA ($m_{i}$) and disease ($d_{j}$) extracted after local and global feature extraction, and $z_{dmd}^{d_{j}}$ represents the semantic features of the disease ($d_{j}$) extracted through the metapath $p_{2}=\left\{\left(d,m,d\right)|m\in M,d\in D\right\}$.

The feature F undergoes three sets of fully connected layers to obtain hierarchical features $H^{0}$, $H^{1}$, and $H^{2}$. Subsequently, a cascaded attention mechanism is employed to supervise inter-layer information interaction and eliminate redundant information.

(15) $$h^{0}=MLP\left(H^{0}\right),h^{1}=MLP\left(H^{1}\right),h^{2}=MLP\left(H^{2}\right)$$

(16) $$h=stack(h^{0},h^{1},h^{2})$$

(17) $$LayerAttention=Softmax(h\times W_{in})$$

(18) $$\tilde{h}=\left(h+\left(h\times LayerAttention\right)\right)\times W_{out}$$

where multi-layer features are unified and stacked along the dimension for computational convenience. Two trainable weight matrices, $W_{in}$ and $W_{out}$, are employed to construct the inter-layer attention matrix and the Softmax activation function is employed to determine the correlation coefficients between layers. $W_{out}$ is used to obtain the multi-layer fused deep semantic representation, denoted as $\tilde{h}$.

Subsequently, a gate-structured feature attention mechanism is employed to establish the correlation between feature elements.

(19) $$FeatureAttention=Softmax(\left(\tilde{h}\times W_{1}\right)\cdot \left(\tilde{h}\times W_{2}\right))$$

(20) $$Out=(\tilde{h}+\left(\tilde{h}\times FeatureAttention\right))$$

The key advantage of cascaded attention lies in its capability to identify which features or feature elements in each layer of the model are crucial for prediction. This mechanism assists the model in better understanding and leveraging the input data, thereby enhancing performance and generalization ability.

## 5. Conclusions

MiRNA plays an important role in various biological processes. When the expression of miRNA is abnormal, these biological processes may be affected, leading to the occurrence of diseases. Therefore, studying the relationship between abnormal expression of miRNA and diseases is of great significance for understanding the pathogenesis of diseases, discovering new therapeutic targets, and developing new treatment methods.

In this work, we propose a novel computational method named MVNMDA for the prediction of associations between miRNA and disease. Specifically, our proposed method utilizes known associations to construct initial node features and subsequently extracts low-dimensional node features through the integration of two homogeneous graph views and one heterogeneous graph view. During the association prediction phase, we employ cascade attention to enhance feature correlations across different layers and reduce noise within the features, ultimately enhancing the predictive performance of the model. To assess the performance of the proposed model, we compare MVNMDA with twelve other computational methods. The results demonstrate that our proposed model achieved the highest AUC and AUPR on both HMDD v2.0 and HMDD v3.2 datasets. To further evaluate the performance of MVNMDA, we conduct case studies on three human diseases, including breast cancer, esophageal cancer, and lung cancer. The results of the case studies indicate the reliability and effectiveness of MVNMDA.

However, despite the progress made by MVNMDA in miRNA-disease association prediction, it still faces challenges from practical issues. Firstly, although the currently known miRNA-disease association data is richer than ever before, the number of unknown associations still far exceeds the experimentally validated known associations. Therefore, accurately selecting reliable negative samples is an urgent problem to be solved. Secondly, the semantic view of MVNMDA relies on manually designed meta-paths to extract semantic information between nodes, which may limit the model’s ability to obtain optimal prediction results. In future research, we plan to introduce an adaptive structure that can autonomously extract features from multi-type node networks in order to improve the performance of the model further. In addition, we are also keenly interested in applying MVNMDA to other related fields, such as protein–protein interactions or drug repositioning [38,39,40].

## Figures and Tables

**Table 1:** The performance comparison of different methods on the HMDD v2.0 dataset.

	Method	AUC (%)	AUPR (%)	Accuracy (%)	Precision (%)	Recall (%)	F1 (%)
**5-fold CV**	AEMDA	91.34	91.27	83.71	81.02	88.05	84.38
	ABMDA	87.60	86.20	80.69	76.69	88.48	82.08
	GBDT-LR	86.18	85.56	80.15	76.90	86.34	81.30
	EDTMDA	91.37	91.60	84.23	83.53	85.35	84.40
	VAEMDA	92.06	92.15	83.82	80.45	89.38	84.66
	NIMCGCN	91.72	91.86	84.38	81.62	88.86	85.04
	ERMDA	90.13	90.43	83.00	80.96	86.39	83.56
	GAEMDA	93.07	92.67	85.22	82.29	89.88	85.88
	SAEMDA	91.54	91.16	83.91	80.65	89.23	84.72
	VGAEMDA	93.85	93.63	87.13	84.45	91.03	87.06
	MINIMDA	94.44	92.96	**87.89**	85.45	**91.60**	**88.39**
	GCNMDA	93.62	92.66	86.51	82.88	91.07	87.22
	MVNMDA	**95.09**	**94.91**	87.68	**86.51**	89.37	87.88
**10-fold CV**	AEMDA	91.59	91.69	84.11	82.13	87.25	84.54
	ABMDA	88.11	87.25	81.15	77.32	88.47	82.43
	GBDT-LR	86.69	86.45	80.77	78.09	85.73	86.18
	EDTMDA	91.93	91.98	84.16	81.38	88.78	84.85
	VAEMDA	92.15	92.04	84.96	82.37	89.08	85.54
	NIMCGCN	91.88	91.92	84.94	83.31	87.63	85.23
	ERMDA	90.52	90.88	83.90	85.28	86.05	84.24
	GAEMDA	93.28	92.80	85.87	83.03	90.22	86.44
	SAEMDA	91.57	91.22	83.99	81.43	88.16	84.61
	VGAEMDA	93.88	93.60	87.18	85.11	90.12	87.53
	MINIMDA	94.06	92.76	87.46	84.08	**92.46**	**88.04**
	GCNMDA	93.59	92.72	86.65	83.14	91.98	87.31
	MVNMDA	**95.04**	**94.88**	**87.87**	**88.28**	87.42	87.81

Bold indicates best performance, underline indicates second best.

**Table 2:** The performance of ablation experiments.

	Local View	Global View	Semantic View	AUC (%)	AUPR (%)	Accuracy (%)	Precision (%)	Recall (%)	F1 (%)
**With Attention**	✓			95.66	95.28	88.84	87.61	90.49	89.02
			✓	94.24	94.27	87.01	87.06	86.96	87.01
	✓	✓		95.84	95.41	89.11	**88.32**	90.15	89.22
	✓		✓	95.82	95.22	88.94	87.49	90.91	89.15
		✓	✓	94.20	94.29	86.92	86.95	86.89	86.91
	✓	✓	✓	**96.13**	**95.99**	**89.42**	88.02	**91.28**	**89.61**
**Without Attention**	✓			95.55	95.24	88.53	87.86	89.41	88.62
			✓	93.82	93.89	86.41	85.57	87.62	86.57
	✓	✓		95.61	95.28	88.59	87.52	**90.01**	88.74
	✓		✓	95.85	95.63	88.88	88.07	89.93	**88.99**
		✓	✓	94.12	94.18	86.89	85.84	88.37	87.07
	✓	✓	✓	**95.87**	**95.68**	**89.01**	**89.40**	88.51	88.95

Bold indicates best performance; underline indicates second best.

**Table 3:** Case study for breast cancer.

Rank	miRNA	Score	Evidence	Rank	miRNA	Score	Evidence
1	hsa-mir-212	0.9410	I,II	16	hsa-mir-130b	0.9090	I,II
2	hsa-mir-142	0.9404	I,II	17	hsa-mir-134	0.9083	I,II
3	hsa-mir-106a	0.9383	I,II	18	hsa-mir-520e	0.9048	I,II
4	hsa-mir-99a	0.9365	I,II	19	hsa-mir-650	0.9022	I,II
5	hsa-mir-130a	0.9346	I,II	20	hsa-mir-503	0.9020	I,II
6	hsa-mir-138	0.9293	I,II	21	hsa-mir-432	0.9016	I,II
7	hsa-mir-372	0.9272	I,II	22	hsa-mir-181c	0.9012	I,II
8	hsa-mir-150	0.9269	I,II	23	hsa-mir-98	0.9008	I,II
9	hsa-mir-378a	0.9267	I,II	24	hsa-mir-216a	0.8999	I,II
10	hsa-mir-184	0.9218	I,II	25	hsa-mir-362	0.8995	I,II
11	hsa-mir-198	0.9214	II	26	hsa-mir-330	0.8981	I,II
12	hsa-mir-517a	0.9206	I,II	27	hsa-mir-449b	0.8968	I,II
13	hsa-mir-381	0.9201	I,II	28	hsa-mir-370	0.8965	I,II
14	hsa-mir-15b	0.9189	I,II	29	hsa-mir-1224	0.8964	I,II
15	hsa-mir-192	0.9180	I,II	30	hsa-mir-483	0.8942	I,II

I denotes verification through HMDD v3.2, and II signifies validation via dbDEMC3.0.

**Table 4:** Case study for esophageal cancer.

Rank	miRNA	Score	Evidence	Rank	miRNA	Score	Evidence
1	hsa-mir-16	0.9332	I,II	16	hsa-mir-182	0.8856	I,II
2	hsa-mir-125b	0.9203	I,II	17	hsa-mir-107	0.8844	I,II
3	hsa-mir-221	0.9167	I,II	18	hsa-mir-9	0.8805	I,II
4	hsa-mir-1	0.9135	I,II	19	hsa-mir-29a	0.8804	I,II
5	hsa-mir-181a	0.9130	I,II	20	hsa-mir-195	0.8776	I,II
6	hsa-mir-133b	0.8991	I,II	21	hsa-mir-125a	0.8775	I,II
7	hsa-mir-222	0.8957	I,II	22	hsa-mir-106a	0.8728	I,II
8	hsa-mir-181b	0.8943	I,II	23	hsa-mir-103a	0.8716	II
9	hsa-mir-106b	0.8935	I,II	24	hsa-mir-199b	0.8688	II
10	hsa-mir-146b	0.8926	I,II	25	hsa-mir-30c	0.8685	I,II
11	hsa-mir-124	0.8905	I,II	26	hsa-mir-93	0.8679	I,II
12	hsa-mir-10b	0.8887	I,II	27	hsa-mir-127	0.8677	I,II
13	hsa-mir-200b	0.8880	I	28	hsa-mir-24	0.8672	I,II
14	hsa-mir-142	0.8875	I,II	29	hsa-mir-30a	0.8670	I
15	hsa-mir-218	0.8863	I,II	30	hsa-mir-17	0.8663	I

I denotes verification through HMDD v3.2, and II signifies validation via dbDEMC3.0.

**Table 5:** Case study for lung cancer.

Rank	miRNA	Score	Evidence	Rank	miRNA	Score	Evidence
1	hsa-mir-16	0.9415	I,II	16	hsa-mir-20b	0.8917	I,II
2	hsa-mir-106b	0.9254	I,II	17	hsa-mir-302b	0.8916	I,II
3	hsa-mir-15a	0.9210	I,II	18	hsa-mir-429	0.8907	I,II
4	hsa-mir-122	0.9180	I,II	19	hsa-mir-194	0.8906	I,II
5	hsa-mir-141	0.9120	I,II	20	hsa-mir-378a	0.8874	I,II
6	hsa-mir-195	0.9112	I,II	21	hsa-mir-193b	0.8856	I,II
7	hsa-mir-15b	0.9098	I,II	22	hsa-mir-129	0.8848	I,II
8	hsa-mir-296	0.9061	I,II	23	hsa-mir-149	0.8830	I,II
9	hsa-mir-99a	0.9059	I,II	24	hsa-mir-302c	0.8776	II
10	hsa-mir-204	0.8996	I,II	25	hsa-mir-23b	0.8769	I,II
11	hsa-mir-152	0.8994	I,II	26	hsa-mir-625	0.8766	I,II
12	hsa-mir-451a	0.8952	I,II	27	hsa-mir-708	0.8723	I,II
13	hsa-mir-151a	0.8949	I,II	28	hsa-mir-342	0.8718	I,II
14	hsa-mir-130a	0.8936	I,II	29	hsa-mir-367	0.8627	I,II
15	hsa-mir-10a	0.8923	I,II	30	hsa-mir-302a	0.8622	II

I denotes verification through HMDD v3.2, and II signifies validation via dbDEMC3.0.

**Table 6:** Median survival of top 6 predictive miRNA in breast cancer (months).

	miRNA-212	miRNA-142	miRNA-106a	miRNA-99a	miRNA-130a	miRNA-138
Low-expression	130.87	129.1	124.53	113.63	129.1	215.2
High-expression	131.97	131.5	131.5	142.23	142.23	115.4

**Table 7:** The statistics for each dataset.

Dataset	miRNAs	Diseases	Known Associations	Unknown Associations	Sparse Ratio
HMDD v2.0	495	383	5430	184,155	0.0286
HMDD v3.2	788	374	8968	285,744	0.0304

## Data Availability

The code is available at https://github.com/yangchen0372/MVNMDA (accessed on 30 December 2023), and the dataset is available for download at https://github.com/chengxu123/MINIMDA (accessed on 30 December 2023).

## Supplementary Materials

The following supporting information can be downloaded at: https://www.mdpi.com/article/10.3390/molecules29010230/s1, Table S1: The prediction results of lung neoplasms; Table S2: The prediction results of breast neoplasms; Table S3: The prediction results of esophageal neoplasms.
